# Combined Effects of Exercise and Broccoli Supplementation on Metabolic and Lipoprotein Biomarkers in Adults with Type 2 Diabetes: A Randomized Controlled Trial

**DOI:** 10.3390/nu17172735

**Published:** 2025-08-23

**Authors:** Maryam Delfan, Masoumeh Gharedaghi, Farzaneh Zeynali, Rawad El Hage, Anthony C. Hackney, Halil İbrahim Ceylan, Ayoub Saeidi, Ismail Laher, Nicola Luigi Bragazzi, Hassane Zouhal

**Affiliations:** 1 Department of Exercise Physiology, Faculty of Sport Sciences, Alzahra University, Tehran 15847-15414, Iran; gharedaghimasum@gmail.com (M.G.); f.zeynali@alzahra.ac.ir (F.Z.); 2 Department of Physical Education, Division of Education, Faculty of Arts and Sciences, University of Balamand, Tripoli P.O. Box 100, Lebanon; 3 Department of Exercise & Sport Science, University of North Carolina, Chapel Hill, NC 27599, USA; 4 Department of Physical Education of Sports Teaching, Faculty of Sports Sciences, Atatürk University, 25240 Erzurum, Türkiye; halil.ceylan@atauni.edu.tr; 5 Department of Physical Education and Sport Sciences, Faculty of Humanities and Social Sciences, University of Kurdistan, Sanandaj 66177-15175, Iran; 6 Department of Anesthesiology, Pharmacology, and Therapeutics, Faculty of Medicine, University of British Columbia, Vancouver, BC V6T 1Z3, Canada; 7 Laboratory for Industrial and Applied Mathematics (LIAM), Department of Mathematics and Statistics, York University, Toronto, ON M3J 1P3, Canada; 8 International Institute of Sport Sciences (2I2S), 35850 Irodouer, France; 9 Laboratoire Optimisation de la Performance Sportive (LR09SEP01) Centre National de la Médecine et des Sciences des Sports, Tunis 1004, Tunisia

**Keywords:** concurrent training, broccoli supplementation, apolipoproteins, diabetes mellitus

## Abstract

**Aim:** To investigate the synergistic effects of exercise training and *Brassica oleracea* var. italica (broccoli sprout) supplementation on Apolipoprotein A-I, B-100, and J levels in men with Type 2 diabetes mellitus (T2DM). **Methods:** Forty-four males with T2DM were randomly assigned to four groups: Control (CG), Supplement (SG), Training (TG), and Training + Supplement (TSG) groups. Participants in the supplement groups (SG and TSG) received 10 g of broccoli supplement after meals for 12 weeks, while those in the training groups (TG and TSG) participated in a structured exercise program (resistance and aerobic), performed three times per week for 12 weeks, at intensities of 60–70% one-repetition maximum (1RM) for resistance training and 60–70% peak oxygen uptake (VO_2peak_) for aerobic training. **Results:** Circulating levels of apolipoproteins improved after 12 weeks in the TSG, TG, and SG groups. However, the TSG group exhibited the most pronounced improvements across metabolic and lipoprotein markers, reflecting an additive effect of both interventions. Specifically, the TSG group demonstrated absolute reductions in ApoB-100 (−48.30 ± 7.20 mg/dL) and ApoJ (−44.05 ± 5.76 mg/dL), along with an increase in ApoA-I (+44.92 ± 6.05 mg/dL). Main effect analysis revealed that exercise training elicited the most substantial improvements across metabolic and lipoprotein markers, with large effect sizes for glucose (*η*^2^*p* = 0.787), insulin (*η*^2^*p* = 0.640), HOMA-IR (*η*^2^*p* = 0.856), ApoA-I (*η*^2^*p* = 0.685), ApoB-100 (*η*^2^*p* = 0.774), ApoJ (*η*^2^*p* = 0.848), and HDL-C (*η*^2^*p* = 0.535). Supplementation showed moderate effects, particularly on HOMA-IR (*η*^2^*p* = 0.370), ApoA-I (*η*^2^*p* = 0.383), and ApoB-100 (*η*^2^*p* = 0.334), supporting an additive but exercise-dominant benefit. The combined intervention group (TSG) showed the most pronounced improvements across all measured outcomes, with large effect sizes for ApoA-I (*η*^2^*p* = 0.883), glucose (*η*^2^*p* = 0.946), insulin (*η*^2^*p* = 0.881), HOMA-IR (*η*^2^*p* = 0.904), and ApoJ (*η*^2^*p* = 0.852). **Conclusions:** The effects of combining training and broccoli sprout supplementation on apolipoprotein levels are likely to result from the activation of two separate pathways, one from training and the other from supplementation. This dual-modality intervention could serve as an effective complementary strategy in managing metabolic and cardiovascular risk factors for individuals with T2DM. However, the magnitude of change induced by the combination of exercise training and broccoli supplementation was largely driven by the training component, with supplementation providing complementary but less consistent benefits.

## 1. Introduction

Type 2 diabetes mellitus (T2DM) is a commonly diagnosed metabolic disease associated with high mortality worldwide [1,2]. Insulin resistance (IR), a hallmark of T2DM, is responsible for hyperglycemia, hyperinsulinemia, oxidative stress, and dyslipidemia [1]. These metabolic disturbances have detrimental effects on the lipid profile in patients with T2DM. Lipoprotein abnormalities, such as high levels of low-density lipoprotein cholesterol (LDL-C) and triglycerides and low levels of high-density lipoprotein cholesterol (HDL-C) contribute to the progression of cardiovascular disease (CVD) [2,3]. Emerging evidence suggests that measuring circulating apolipoproteins may offer greater predictive value than traditional lipid parameters in assessing the risk of T2DM, thereby providing novel insights not only into disease pathophysiology but also into potential pharmacological targets for the development of more precise, mechanism-based therapies and interventions [2].

Apolipoproteins are a class of proteins that play crucial roles in the structure, function, metabolism, and regulation of lipoproteins [4]. Among the various apolipoproteins, Apolipoprotein A (ApoA-I), Apolipoprotein B100 (ApoB-100), and Apolipoprotein J (ApoJ) have been extensively studied due to their association with cardiovascular health [5]. ApoA-I is the most abundant protein component of HDL and plays a role in glucose uptake by the skeletal muscle and heart [6]. Reduced plasma levels of ApoA-I in T2DM may be due to decreased responsiveness of the ApoA-I gene to insulin or the reduction in plasma HDL levels [7]. ApoB-100 is a significant structural component of atherogenic lipoproteins, such as LDL, very-low-density lipoproteins (VLDL), and intermediate-density lipoproteins (IDL) [8]. Increased levels of ApoB-100 are associated with the development of atherosclerosis plaques [9]. Another important apolipoprotein is ApoJ, also known as clusterin [10]. It interacts with specific receptors, including the HDL-C receptor, LDL-related protein 2 (LRP2), ApoE receptor 2 (ApoER2), and the VLDL receptor (VLDLR). ApoJ is involved in the pathophysiology of several metabolic conditions, including obesity, T2DM, and CVD [5,10]. Increased circulating levels of ApoJ have been reported in individuals with T2DM, obesity, and systemic inflammation—conditions commonly characterized by insulin resistance [11].

Non-pharmacological strategies for managing T2DM emphasize lifestyle interventions, such as a healthy diet and regular physical activity [12,13]. Herbal supplements also have a role in the management of T2DM [14]. Broccoli and broccoli sprouts contain bioactive compounds, including antioxidant vitamins, minerals such as selenium, phenolic compounds, and glucosinolates like indole-3-carbinol [15]. A four-week intake of broccoli (10 g/d) reduces serum insulin levels and improves the homeostatic model assessment for insulin resistance (HOMA-IR) in individuals with T2DM [16]. Sulforaphane (SFN), another key compound in broccoli, reduces lipid peroxidation, enhances total antioxidant capacity, and decreases oxidative stress in individuals with T2DM by activating antioxidant pathways [17]. Furthermore, SFN improves muscle strength, function, and exercise capacity while protecting muscles from oxidative damage and inflammation [18]. Additionally, broccoli supplementation has been shown to reduce exercise-induced muscle soreness and lower oxidative stress indicators in individuals with T2DM [19].

Aerobic exercise improves peak oxygen uptake (VO_2peak_), IR, glycated hemoglobin (HbA1c), and triglyceride levels [20]. Resistance exercise decreases fat mass and blood pressure, increases muscle strength, promotes muscle rehabilitation, and enhances glucose transporter type 4 (GLUT-4) expression in individuals with T2DM and obesity [21,22]. Combining aerobic and resistance exercises may have a synergistic effect on T2DM and obesity [22,23]. It can increase HDL-C levels and decrease plasma ApoJ levels by reducing body weight, total fat mass, and improving skeletal muscle mass in individuals with T2DM [24].

In short, exercise can improve various health parameters in patients with T2DM [25]. Additionally, studies indicate that taking dietary supplements can have a positive impact on overall physical health [17,26]. However, only a few studies have examined the effect of combining exercise and nutritional supplements on patients with T2DM [27]. Some studies suggest that combining dietary supplements with regular exercise could help maximize their synergistic effects [27]. Nonetheless, there is insufficient research to demonstrate how the combination of exercise and dietary supplements affects T2DM. While previous studies have independently examined the effects of broccoli supplementation and combined training on apolipoproteins [17,24], the synergistic effects of these two interventions remain unclear. For example, in postmenopausal women with T2DM, twelve weeks of combined aerobic and resistance training significantly reduced circulating levels of ApoJ and improved indices of IR [27]. Separately, a previous review study reported that broccoli sprout supplementation (rich in SFN) decreased oxidative stress, lipid peroxidation, serum triglycerides, IR, and inflammatory markers in patients with T2DM [17]. Therefore, the main objective of this study was to investigate the combined effects of broccoli sprout supplementation and exercise training on circulating levels of ApoA-I, ApoB-100, and ApoJ in men with T2DM. A secondary objective was to assess the impact of the intervention on glycemic markers, such as fasting blood glucose (FBG) and HOMA-IR, to explore potential metabolic interactions beyond lipid-related outcomes. Due to limited access to eligible female participants and to minimize sex-related biological variability in metabolic and hormonal responses, the present study was conducted solely on males.

## 2. Methods

### 2.1. Participants

**Figure 1:**
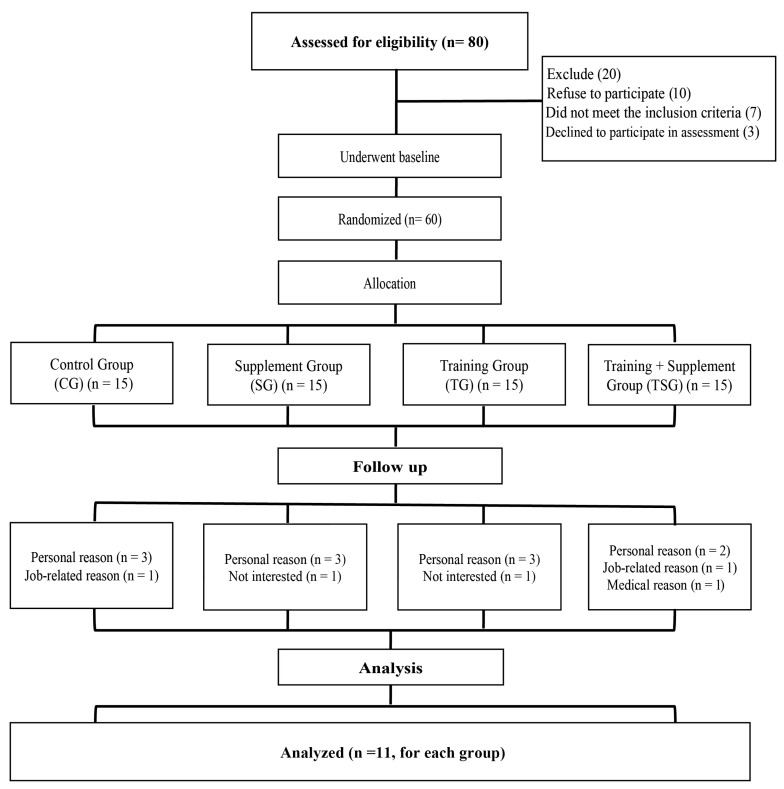
Pictorial flowchart of the experimental design of the present study.

A total of 80 individuals volunteered to participate in the study following extensive recruitment efforts conducted across public spaces, research laboratories, sports clubs, and social media platforms. Of these, 20 individuals were excluded for not meeting the established inclusion criteria (Figure 1). All participants underwent medical screening by a certified cardiologist to confirm their health status and suitability for participation.

This study was a 12-week randomized controlled trial involving four intervention groups. Participants were assigned to one of the four groups using simple randomization. The randomization sequence was generated using SPSS version 26. To ensure allocation concealment, group assignments were performed by an independent researcher who was not involved in delivering the intervention or assessing outcomes.

The study protocol was reviewed and approved by the Ethics Committee of the Sport Sciences Research Institute, Tehran, Iran (Ethics code: IR.SSRC.REC.1401.142), and all procedures were conducted in accordance with the most recent version of the Declaration of Helsinki [28].

**Inclusion criteria:** Participants had to be males between the ages of 40 and 60, diagnosed with T2DM according to the American Diabetes Association guidelines, have had T2DM for more than two years, have FBG levels of 126 mg/dL or higher, and be receiving oral hypoglycemic medications.

**Exclusion criteria:** Participants with cardiac pathologies, hypertension (defined as blood pressure exceeding 160/90 mmHg), musculoskeletal diseases, proliferative diabetic retinopathy, advanced nephropathy, FBG levels exceeding 270 mg/dL, those receiving insulin injections, and individuals taking lipid- and blood-pressure-lowering medications were excluded from the study. This exclusion criterion was implemented to minimize the confounding effects of pharmacological interventions and to ensure that any observed changes could be attributed to the study intervention. All participants were under regular medical supervision, and those requiring statin or antihypertensive therapy according to clinical guidelines were excluded from participation due to ethical considerations.

### 2.2. Experimental Design

#### 2.2.1. Baseline Evaluations

All participants attended a familiarization session one week before the initiation of the training protocols. During this session, the study procedures were clearly explained, and participants completed the Physical Activity Readiness Questionnaire (PAR-Q). In addition, each participant’s height and weight were measured to calculate their body mass index (BMI).

#### 2.2.2. Study Design

Following the baseline evaluations, the 60 eligible participants were randomly allocated to four study groups of 15 individuals each: the Control Group (CG), the Supplement Group (SG), the Training Group (TG), and the Training + Supplement Group (TSG).

Participants in the intervention groups were closely monitored for adherence. Following the American College of Sports Medicine’s guideline of at least 80% adherence as adequate participation in exercise programs [29], individuals who missed more than seven of the 36 training sessions were excluded from the analysis. Supplement adherence was defined as consuming at least 80% of the assigned doses. Therefore, the adherence rate among the remaining participants in the TG, SG, and TSG groups was considered to be 100%.

Furthermore, during the course of the protocol, several participants were excluded from the study due to either personal reasons—such as voluntary withdrawal, scheduling conflicts, or difficulty complying with study instructions—or medical reasons, including previously undisclosed conditions or newly arising health issues. These exclusions were made to protect participant safety and to ensure the validity and consistency of the data collected.

During the third session, each group received comprehensive instructions on how to perform their respective training protocols. Following baseline measurements, both training groups (TG and TSG) commenced a structured 12-week exercise program. During the study, participants were asked to carefully record their food intake over three consecutive days, including two weekdays and one weekend day. These records calculated total daily caloric intake (in kilocalories) and the amounts of carbohydrates, fats, and proteins (in grams). Dietary data were analyzed using Diet Analysis Plus software, version 10 (Cengage, Boston, MA, USA) (Table 1). Those assigned to the control and supplement groups were advised to continue their daily routines throughout the study. All measurements were taken roughly at the same time each day (with a deviation of about 1 h) and under consistent environmental conditions (approximately 20 °C and ~55% humidity).

#### 2.2.3. Peak Oxygen Uptake, Blood Pressure, Heart Rates, and One-Repetition Maximum Assessments

A modified Bruce protocol using a motorized treadmill (Pulsar 3p, H/p/Cosmos, Nussdorf-Traunstein, Germany) with a gas analyzer system (Metalyzer 3B Analyzer, Cortex Biophysik, Leipzig, Germany) was used to estimate VO_2peak_ [30]. Systolic and diastolic blood pressure measurements were obtained using an automated device (Omron M6 Comfort HEM-7221-E, Omron Healthcare, Kyoto, Japan). Simultaneously, heart rate (HR, beats per minute) was continuously measured using a heart rate monitoring device (Polar Electro, Espoo, Finland). The intensity of aerobic exercise was determined based on the individual’s VO_2peak_. A 1-repetition maximum (1-RM) method was used to assess muscle strength for all strength exercises; the 1-RM measurement occurred during the final familiarization session. Patients engaged in a 5-min warm-up before the exercises to prevent injuries, and they were instructed to lift the maximum amount of weight they could manage for 6–8 repetitions for each exercise. Subsequently, the 1-RM value was calculated using the Brzycki formula [31].

#### 2.2.4. Training Protocols

The exercise training sessions for patients with T2DM (TG and TSG groups) were closely supervised by exercise physiologists and endocrinologists, ensuring professional oversight. These sessions consisted of both aerobic and resistance exercises, meticulously planned in terms of volume and intensity for patients with T2DM [32]. Each session began with a 5- to 10-min warm-up period, which included walking and running on a treadmill at 50–60% of the participants’ maximum heart rate (HR_max_), followed by 45 min of resistance training that involved nine specific movements targeting both the upper and lower body. These movements included the leg press, knee flexion, knee extension, chest press, lat pull-down, abdominal crunches, biceps curls, triceps press down, and shoulder press. The resistance exercises were performed at 60–70% of the patients’ 1-RM. In addition to resistance training, aerobic exercises were incorporated, lasting 30 min and maintained at 60–70% of the patients’ VO_2peak_. These aerobic sessions were conducted on a motorized treadmill (Pulsar 3p, H/p/Cosmos, Nussdorf-Traunstein, Germany). The duration of aerobic exercise gradually increased from 10 min in the first week to 30 min by the eighth week and remained at this level throughout the study (Table 2). The 1-RM and VO_2peak_ tests were repeated every four weeks to adhere to the principle of overloading.

#### 2.2.5. Supplementation of Broccoli Sprout and Placebo

Neither the participants nor the researchers were aware of the supplement assignments. This study used a broccoli sprout powder supplement (Cyvex Nutrition Company, Irvine, CA, USA). The dosage of SFN administered was 225 μmol per 10 g/d of the broccoli sprout powder supplement. The SG and TSG group participants were instructed to consume one packet of the broccoli sprout supplement (10 g) daily for 12 weeks [33,34]. Comparable amounts of placebo were also provided for both the CG and TG. The placebo was formulated using a mixture of 5 g of cornstarch powder and spinach powder (in a 10:1 ratio) and prepared to resemble the appearance of the broccoli sprout supplement. Supplement adherence was defined as participants consuming at least 80% of their assigned doses (i.e., 67 packets).

#### 2.2.6. Biochemical Analysis

All testing was conducted under standard conditions between 8 a.m. and 10 a.m. Blood samples were taken (after 12 h of fasting) from the right arm 48 h before the first exercise session and 48 h after the last session. Blood samples were collected in EDTA tubes for plasma isolation, centrifuged at 3500 rpm for 15 min, and stored at −80 °C. Glucose assay kits measured glucose levels with a sensitivity of 1 mg/dL. Insulin levels were assessed using a radioimmunoassay kit (Diagnostic Systems Laboratories, Webster, TX, USA). The HOMA-IR was used to estimate the insulin resistance index, using the following formula: fasting plasma glucose (mmol/L) × fasting plasma insulin (μU/mL)/22.5 [35]. Plasma triglycerides, LDL-C, HDL-C, and total cholesterol (TC) levels were measured using a standard biochemical analyzer (DAX 96; Bayern Diagnostics, Milan, Italy). The plasma concentrations of ApoA-I, ApoB-100, and ApoJ were assessed with ELISA kits (Zellbio, Lonsee, Germany). The coefficients of variation were 1.2% for glucose, 1.8% for insulin, 2.9% for HOMA-IR, and less than 5% for HDL-C, LDL-C, TC, and triglycerides.

#### 2.2.7. Statistical Analysis

A power analysis was conducted using the software G*Power Version 3.1.9.6 (Kiel, Germany) to calculate the sample size. The input parameters were an alpha level of 0.05 and 95% statistical power. The study by Delfan et al. [36] was used as a reference, as it employed a similar study design. From this study, we extracted the effect size measure, Cohen’s f = 0.88, for the outcome insulin and included it in our a priori power analysis. The results of the power analysis indicated that a total of 12 participants per group would be needed to achieve significant group-by-time interactions. To account for potential dropouts, 15 participants were initially recruited in each group. Statistical analysis was performed using GraphPad Prism software version 8.4.0 (GraphPad Software, Boston, MA, USA). The data were initially assessed for their distribution using the Kolmogorov–Smirnov test, and the results were analyzed with three-way ANOVA with repeated measures (2 × 2 × 2; supplementation [broccoli vs. placebo] × training [yes vs. no] × time [baseline vs. end]). The partial eta squared for main effects was calculated from the ANOVA (*η*^2^*p*) and was interpreted as follows: 0.01 = small effect, 0.06 = medium effect, and 0.14 = large effect [37]. When a significant interaction was found, the Bonferroni post hoc test was performed to determine differences between groups [SG, TG, TSG, and CG]. Data are shown as mean ± SD; *p* < 0.050 was considered statistically significant.

## 3. Results

### 3.1. Anthropometry

A total of 16 participants (n = 4 per group) withdrew from the study due to medical issues, work-related constraints, or a lack of sustained interest, resulting in 44 participants remaining in the study (n = 11 per group) for the final analysis. The groups were comparable in terms of age, ethnicity, duration of diabetes, and medication use. There were no differences (*p* > 0.050) in body mass and BMI between the study groups at the beginning of the study (Table 3). Weight reductions were evident in both training groups compared to the pre-test measurements (*p* < 0.0007). Additionally, BMI alterations were observed from baseline to 12 weeks in both the TG and TSG groups; however, significance was evident only in the TSG group (*p* = 0.021).

### 3.2. Glucose, Insulin, and HOMA-IR

**Figure 2:**
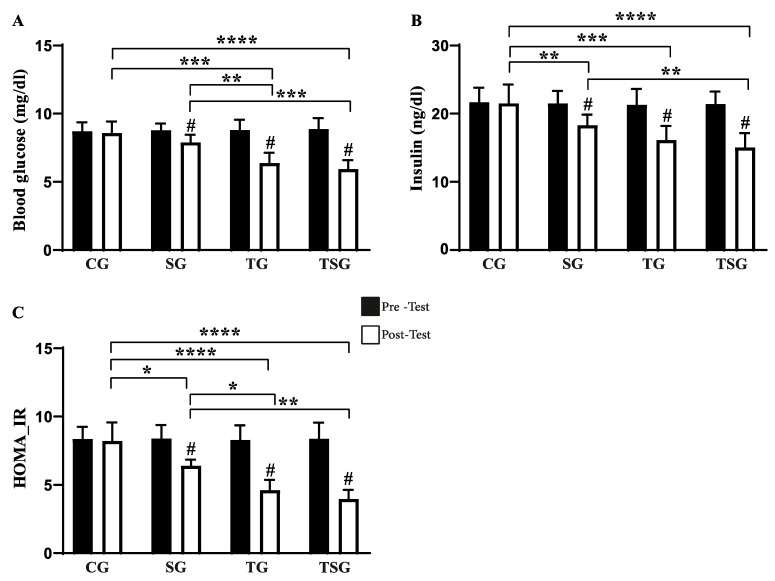
Pre-test and post-test intervention values of (**A**) fasting glucose, (**B**) insulin, and (**C**) HOMA-IR in CG: Control Group, SG: Supplement Group, TG: Training Group, TSG: Training + Supplement Group. # indicates a significant difference from baseline. * Significant differences: * *p* < 0.05, ** *p* < 0.01, *** *p* < 0.001, **** *p* < 0.0001.

There was a main effect of training (*p* < 0.001, *η*^2^*p*: 0.787) and time (*p* < 0.001, *η*^2^*p*: 0.946) on blood glucose concentration, while the main effect of supplementation did not reach statistical significance for this variable (*p* = 0.075, *η*^2^*p*: 0.283). The post hoc analysis revealed that despite comparable values before the interventions across all four groups, only the SG, TG, and TSG reduced blood glucose concentrations after the intervention (all *p* < 0.050, Figure 2A). At the end of the interventions, blood glucose concentration in the CG were higher than in the TG and TSG (*d* = 0.96, *p* < 0.01 and *d* = 1.19, *p* < 0.01, respectively) and those in the SG were also higher than in the TG and TSG (*d* = 0.93, *p* < 0.01 and *d* = 1.16, *p* < 0.01, respectively). However, there were no differences in blood glucose concentration between the TG and TSG at the end of the interventions (*d* = 0.87, *p* = 0.065).

There was a main effect of training (*p* = 0.002, *η*^2^*p*: 0.640) and time (*p* < 0.001, *η*^2^*p*: 0.881) on insulin concentration, while the main effect of supplementation did not reach statistical significance for this variable (*p* = 0.051, *η*^2^*p*: 0.330). The post hoc analysis revealed that despite comparable values before the interventions across all four groups, only the SG, TG, and TSG reduced insulin concentrations after the intervention (all *p* < 0.050, Figure 2B). At the end of the interventions, insulin concentration in the CG was higher than in the SG, TG, and TSG (*d* = 2.92, *p* < 0.01, *d* = 3.95, *p* < 0.01, and *d* = 3.71, *p* < 0.01, respectively) and in the SG it was higher than in the TSG (*d* = 2.75, *p* < 0.01), without differences between the TG and TSG (*d* = 0.96, *p* = 0.252).

There was a main effect of supplementation (*p* = 0.036, *η*^2^*p*: 0.370), training (*p* < 0.001, *η*^2^*p*: 0.856), and time (*p* < 0.001, *η*^2^*p*: 0.904) onHOMA-IR. The post hoc analysis revealed that despite comparable HOMA-IR values before the interventions across all four groups, only the SG, TG, and TSG reduced HOMA-IR after the intervention (all *p* < 0.050, Figure 2C). At the end of the interventions, HOMA-IR in the CG was higher than in the SG, TG, and TSG (*d* = 1.36, *p* < 0.05, *d* = 1.41, *p* < 0.01 and *d* = 1.36, *p* < 0.01, respectively) and in the SG it was higher than in the TG (*d* = 0.65, *p* < 0.05) and in the TSG (*d* = 1.03, *p* < 0.01), without differences between the TG and TSG (*d* = 0.71, *p* = 0.084).

### 3.3. Lipid Profile

**Figure 3:**
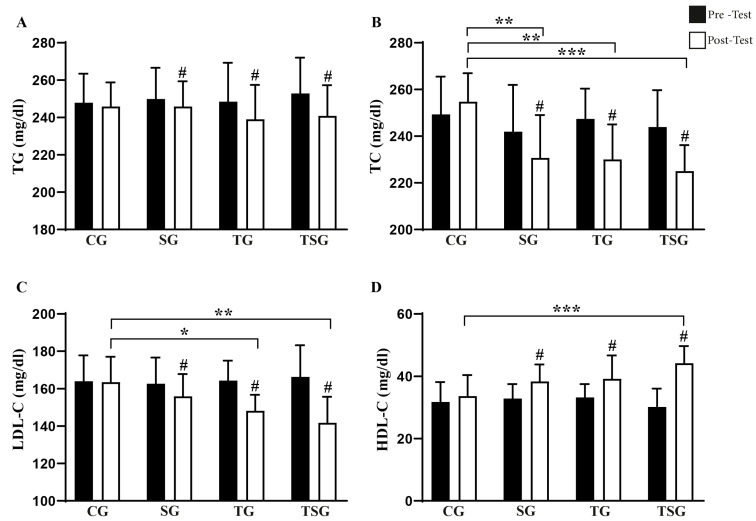
Pre-test and post-test values for lipid profile. (**A**) TG: Triglycerides. (**B**) TC: Total Cholesterol (**C**) LDL-C: Low-Density Lipoprotein Cholesterol and (**D**) HDL-C: high-density lipoprotein cholesterol in CG: Control Group, SG: Supplement Group, TG: Training Group, TSG: Training + Supplement Group. # indicates a significant difference from baseline. Significant differences: * *p* < 0.05, ** *p* < 0.01, *** *p* < 0.001.

There was a main effect of time (*p* < 0.001, *η*^2^*p*: 0.929) on TG concentration with no effect of training (*p* = 0.661, *η*^2^*p*: 0.020) or supplementation for this variable (*p* = 0.694, *η*^2^*p*: 0.016). The post hoc analysis revealed that TG values before the interventions were similar across all four groups, but only the SG, TG, and TSG reduced TG after the intervention (all *p* < 0.050, Figure 3A). However, post-intervention values of TG were similar across all four groups.

There was a main effect of supplementation (*p* = 0.039, *η*^2^*p*: 0.360) and time (*p* < 0.001, *η*^2^*p*: 0.826) on TC concentration, while the main effect training did not reach statistical significance for this variable (*p* = 0.179, *η*^2^*p*: 0.173). The post hoc analysis revealed that TG values before the interventions were similar across all four groups but only the SG, TG, and TSG reduced TC after the intervention (all *p* < 0.050, Figure 3B). At the end of the interventions, TG in the CG was higher than in the SG (*d* = 1.95, *p* < 0.01), TG (*d* = 2.01, *p* < 0.01), and TSG (*d* = 2.31, *p* < 0.01).

There was a main effect of time (*p* < 0.001, *η*^2^*p*: 0.945) on LDL-C concentration with no effect of training (*p* = 0.183, *η*^2^*p*: 0.170) or supplementation for this variable (*p* = 0.256, *η*^2^*p*: 0.127). The post hoc analysis revealed that LDL-C values before the interventions were similar across all four groups but only the SG, TG, and TSG reduced LDL-C after the intervention (all *p* < 0.050, Figure 3C). At the end of the interventions, LDL-C in the CG was higher than in the TG (*d* = 1.56, *p* < 0.05) and TSG (*d* = 2.12, *p* < 0.01).

There was a main effect of training (*p* = 0.007, *η*^2^*p*: 0.535) and time (*p* < 0.001, *η*^2^*p*: 0.939) on HDL-C concentration, while the main effect of supplementation did not reach statistical significance for this variable (*p* = 0.338, *η*^2^*p*: 0.092). The post hoc analysis revealed that HCL-C values before the interventions were similar across all four groups but only the SG, TG, and TSG increased HDL-C after the intervention (all *p* < 0.050, Figure 3D). At the end of the interventions, HDL-C in the CG was only lower than in the TSG (*d* = 8.76, *p* < 0.01).

### 3.4. ApoA-I, ApoB-100, and ApoJ

**Figure 4:**
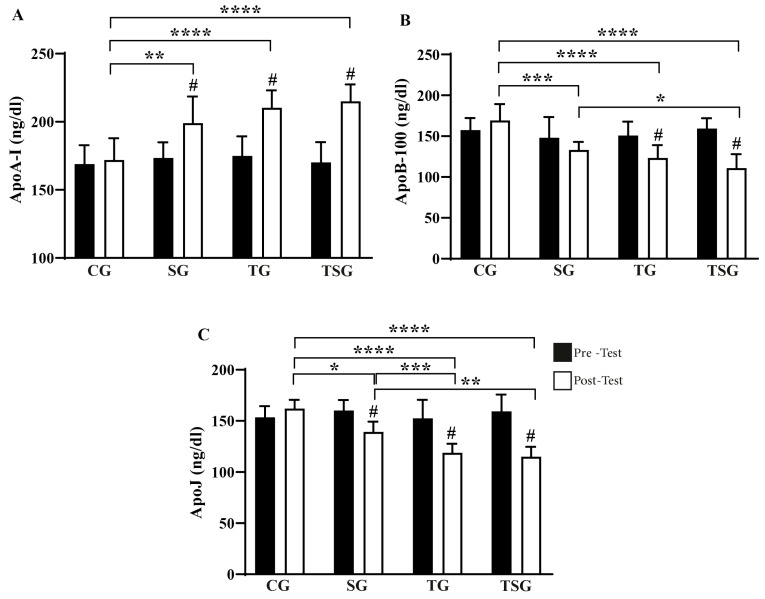
Pre-test and post-test values (mean ± SD) for (**A**) ApoA-I, (**B**) ApoB-100, and (**C**) ApoJ in CG: Control Group, SG: Supplement Group, TG: Training Group, TSG: Training + Supplement Group. # indicates a significant difference from baseline. * Significant differences: * *p* < 0.05, ** *p* < 0.01, *** *p* < 0.001, **** *p* < 0.0001.

There was a main effect of supplementation (*p* < 0.032, *η*^2^*p*: 0.383), training (*p* < 0.001, *η*^2^*p*: 0.685), and time (*p* < 0.001, *η*^2^*p*: 0.883) on ApoA-I concentration. The post hoc analysis revealed that despite comparable values before the interventions across all four groups, only the SG, TG, and TSG increased ApoA-I concentrations after the intervention (all *p* < 0.050, Figure 4A). At the end of the interventions, ApoA-I in the CG were than lower in the SG (*d* = 1.67, *p* < 0.01), TG (*d* = 2.45, *p* < 0.01), and TSG (*d* = 1.48, *p* < 0.01), without differences among these three groups.

There was a main effect of supplementation (*p* < 0.049, *η*^2^*p*: 0.334), training (*p* < 0.001, *η*^2^*p*: 0.774), and time (*p* < 0.001, *η*^2^*p*: 0.684) on ApoB-100 concentration. The post hoc analysis revealed that despite comparable values before the interventions across all four groups, only the TG and TSG reduced ApoB-100 concentrations after the intervention (all *p* < 0.050, Figure 4B). At the end of the interventions, ApoB-100 in the CG were higher than in the SG (*d* = 1.80, *p* < 0.01), TG (*d* = 1.68, *p* < 0.01), and TSG (*d* = 2.57, *p* < 0.01). Additionally, ApoB-100 concentrations in the SG were higher than in the TSG (*d* = 1.75, *p* = 0.02).

There was a main effect of training (*p* < 0.001, *η*^2^*p*: 0.848) and time (*p* < 0.001, *η*^2^*p*: 0.852) on ApoJ concentration, while the effect of supplementation did not reach statistical significance (*p* = 0.103, *η*^2^*p*: 0.244) The post hoc analysis revealed that despite comparable values before the interventions across all four groups, only the SG, TG, and TSG reduced ApoJ concentrations after the intervention (all *p* < 0.050, Figure 4C). At the end of the interventions, ApoJ concentrations in the CG were higher than in the SG (*d* = 0.85, *p* < 0.05), TG (*d* = 1.34, *p* < 0.01) and TSG (*d* = 2.51, *p* < 0.01). Additionally, ApoJ concentrations in the SG were higher than in the TG (*d* = 1.68, *p* = 0.03) and TSG (*d* = 1.75, *p* < 0.01).

## 4. Discussion

Our study demonstrated that a 12-week intervention combining exercise training and broccoli sprout supplementation elicited significant improvements in key metabolic and lipoprotein biomarkers in men with T2DM. Notably, the three-way ANOVA revealed robust main effects of training across nearly all variables, including glucose, insulin, HOMA-IR, ApoA-I, ApoB-100, ApoJ, and HDL-C, with large effect sizes (*η*^2^*p* ranging from 0.535 to 0.856). Broccoli sprout supplementation also contributed to improvements, particularly in HOMA-IR, TC, ApoA-I, and ApoB-100, though its effects were generally more modest. The combined intervention group (TSG) consistently showed the greatest magnitude of change, suggesting additive benefits in some variables. However, the marginal differences between the TSG and TG groups imply that the majority of the observed benefits may be attributed to the training component. These findings underscore the dominant role of structured exercise training in modulating metabolic health in T2DM, while also highlighting the complementary value of broccoli sprout supplementation.

Circulating levels of ApoA-I increased in the SG, TG, and TSG compared to the CG, with the most significant increases observed in the TG and TSG. This aligns with previous findings that combined exercise training increased ApoA-I levels [38]. Although we did not directly measure the underlying molecular mediators, we hypothesize that this change in ApoA-I levels could result from combined training elevating ATP-binding cassette transport (ABC) proteins, including the A group (ABCA) and ABC group (ABCG), which can increase *ApoA* gene expression [39]. Another study reported that a 17-week diet supplemented with 10% (*w*/*w*) broccoli florets or broccoli stalks in male mice (7–8 weeks old) increased the levels of peroxisome proliferator-activated receptor α (PPARα) and adenosine monophosphate-activated protein kinase (AMPK) [40]. While we did not assess PPARα or AMPK activation in our study, their involvement is proposed as a possible mechanism for the observed changes and warrants investigation in future research. PPARα activation has been reported to increase liver *ApoA-I* mRNA levels [41]. The regulatory function of PPARα on gene expression of HDL apolipoproteins may have important clinical implications [41]. Higher ApoA-I levels are inversely associated with cardiovascular risk, indicating a protective effect, particularly in individuals with T2DM. From a clinical perspective, the increase in ApoA-I levels observed in our study approaches the upper reference range for men (above 120 mg/dL), a threshold associated with enhanced reverse cholesterol transport and reduced cardiovascular risk. This suggests that the elevation in ApoA-I may be clinically beneficial, especially for participants with low baseline levels [42].

Circulating levels of ApoB-100 decreased in the SG, TG, and TSG compared to the CG, with decreases in the TSG being statistically significant compared to the SG. A study investigating the secretion of ApoB-100 from HepG2 cells treated with indole-3-carbinol reported that it could inhibit the secretion of ApoB-100. This improvement may be explained by the finding that it suppresses the protein expression of sterol regulatory element-binding protein (SREBP)-1. A reduction in lipid synthesis caused by indole-3-carbinol could potentially decrease the amount of lipid substrates required for ApoB-100 assembly [15]. Research has shown that 24 weeks of combined training (aerobic and resistance training) decreased plasma ApoB-100 concentrations in middle-aged obese women, indicating the positive effects of these training methods in preventing CVD [38]. This finding may be related to increased insulin sensitivity, as findings suggest that combined training (aerobic and resistance training) increased IRS-1 expression by 90% [22]. The increase in IRS-1 expression leads to improved insulin signaling through the MAPK (mitogen-activated protein kinase) activation pathway, and a reduction in MTTP (microsomal triglyceride transfer protein) expression, which inhibits the release of ApoB-100 [43]. MTTP, as a transporter of triglycerides, transfers triglycerides, phospholipids, and cholesterol to ApoB-100 in lipidation. ApoB-100 would be degraded if not subjected to lipidation. Therefore, insulin likely degrades ApoB-100 through the action of MTTP [44]. Clinically, ApoB-100 is a well-established marker of the atherogenic particle number, and its reduction is considered a strong predictor of decreased cardiovascular risk. The observed decrease in ApoB-100 levels is clinically significant compared to reference values [45].

We also found reduced ApoJ levels in the TG and TSG compared to the SG and CG. An increase in adipocyte-derived ApoJ in the circulation is a marker of IR [5]. A recent study reported that consuming broccoli florets or broccoli stalks increased AMPK and PPARα levels in mice [40]. PPARα levels are associated with increases in LRP2 expression, which belongs to the low-density lipoprotein receptor (LDLR) protein family. Furthermore, PPARα agonists induce significant increases in *LRP2* mRNA and protein expression. LRP2 is an endocytic cell surface receptor that belongs to the LDL receptor family [46] and, as a potential receptor for ApoJ, plays a crucial role in patients with T2DM. As a result, if muscle LRP2 is increased, ApoJ levels decrease in the serum [47]. However, since we did not assess AMPK, PPARα, or LRP2 levels, these findings remain speculative and should be interpreted as hypotheses for future research. Additionally, adipocytes are increasingly recognized as essential sites for the secretion of ApoJ. Combined training decreases intra-abdominal fat and ApoJ levels more effectively than food restriction [38]. Our findings in obese males support previous findings that decreases in circulating ApoJ levels induced by exercise are associated with improvements in IR in T2DM [24]. A prior study demonstrated that ApoJ impairs insulin signaling by decreasing Akt phosphorylation and upregulating genes related to gluconeogenesis, indicating that enhanced insulin sensitivity may be linked to lower ApoJ concentrations [5,47]. Although reference ranges for ApoJ are less well-established clinically, elevated ApoJ has been associated with IR and metabolic dysfunction. Therefore, the reduction observed in ApoJ levels suggests a potentially beneficial adaptive response. This may have clinical implications for improving insulin sensitivity in patients with T2DM [11]. In this context, it is plausible that the reduction in ApoJ observed in our study may be due to reduced adiposity, improved insulin sensitivity, or a combination of both.

Our study also demonstrates decreases in insulin and HOMA-IR levels in the SG, TG, and TSG, with the most significant improvements observed in the training groups. Combined training appears to be more effective in enhancing insulin and glucose uptake in individuals with T2DM, possibly due to the distinct mechanisms of combined training [48,49].

Our findings also demonstrate improvements in the lipid profiles in the experimental groups, with significant differences in HDL-C and LDL-C levels between the TSG and CG. Broccoli sprout supplementation lowers plasma LDL-C concentrations by modulating cellular and tissue redox balance, enhancing AMPK signaling, and inhibiting the phosphoinositide 3-kinase (PI3K) pathway [50]. Moreover, aerobic exercise and weight loss reduce blood cholesterol, LDL-C, triglycerides, and HDL-C [51].

There was weight loss in the TG and TSG groups after 12 weeks, while body mass did not differ between the CG and SG groups. Additionally, Murashima et al. (2004) reported that consuming fresh broccoli (100 g/day) for one week decreased TC and LDL-C while significantly increasing HDL-C in twelve healthy subjects [52]. Furthermore, the consumption of broccoli sprout powder by patients with T2DM has been reported to improve their lipid profiles [33], consistent with our results. Moreover, combined training (aerobic and resistance exercise) improves lipid profiles in individuals with T2DM [20]. Increasing insulin sensitivity and improving insulin signaling may be a mechanism for changes in lipid profiles following combined training [53]. We hypothesized that combining exercise with broccoli sprout supplementation would result in greater improvements in apolipoprotein levels compared to either intervention alone. However, except for ApoA-I, we found that the TSG exhibited greater changes in ApoB-100 and ApoJ levels compared to the SG and CG, but not the TG. Interestingly, however, when the predefined contrasts were performed, we found that the combined group elicited significantly greater improvements in all apolipoprotein levels compared to the following groups: (1) CG, (2) SG, and (3) TG, as well as the comparison of the CG, TG, and SG.

Taken together, consuming broccoli sprouts and engaging in combined training can synergistically benefit circulating ApoB-100, ApoJ, glucose, insulin, and HOMA-IR levels, beyond that of supplementation or training alone. Although the statistical analysis did not demonstrate significant differences between the TSG and TG, the percentage changes in key variables were greater in the TSG. This trend suggests that combining training and broccoli sprout supplementation may modulate apolipoprotein levels through distinct biological pathways, potentially resulting in synergistic effects.

Moreover, the robust contrast analysis, taking into consideration the grand means of the specified contrasts, highlighted the superiority of the combined intervention group. Nevertheless, a definitive conclusion regarding a synergistic interaction cannot be drawn without further studies with larger sample sizes and mechanistic investigations to determine whether these interventions exert synergistic effects and elucidate the underlying biological mechanisms.

### Study Limitations

Our study cannot be generalized, as young men and women were excluded from patient recruitment. Additionally, we did not monitor dietary intake and energy expenditure, which can potentially have significant impacts on various physiological outcomes. This lack of dietary control may have introduced inter-individual variability in metabolic responses, particularly in glucose- and lipid-related parameters. We did not measure muscle mass or the percentage and distribution of body fat. Additionally, some effect sizes in our study were not very robust, possibly due to the small sample size (n = 11) in each group. This small sample size may have reduced the statistical power and increased the risk of Type II error, potentially masking subtle but clinically meaningful effects. Future studies with larger sample sizes are recommended to confirm these results. Moreover, the SFN content of the broccoli sprout supplement was not independently verified in our laboratory. It was solely based on the manufacturer’s information, which may limit the precision of our interpretation regarding its bioactivity. Lastly, our study included a homogenous group of individuals diagnosed with T2DM, which limits our interpretations as this condition is phenotypically heterogeneous. Future research should aim to include women, younger individuals, and other age groups to enhance the generalizability and external validity of the findings.

One limitation of this study is the absence of prior registration in a public clinical trial registry. Although the study design fulfills the criteria for a randomized controlled trial, registration was not completed before participant enrollment due to limited awareness of international registration requirements at the time. We acknowledge this limitation and are committed to ensuring prospective registration of all future clinical trials in accordance with ICMJE and WHO standards.

## 5. Conclusions

This study highlights the efficacy of combining structured exercise training with broccoli sprout supplementation as a multifaceted strategy to improve metabolic and cardiovascular biomarkers in men with T2DM. The intervention led to significant improvements in ApoA-I, ApoB-100, ApoJ, insulin, glucose, HOMA-IR, and HDL-C, with training emerging as the dominant contributor to these effects. While supplementation alone showed moderate benefits, its combination with exercise amplified the overall impact, particularly on apolipoprotein and insulin resistance markers. From a practical standpoint, these findings support the integration of supervised aerobic and resistance training into standard diabetes care, with broccoli sprout supplementation serving as a safe and accessible adjunct. Clinicians and exercise professionals may consider recommending this dual-modality approach to enhance lipid metabolism, glycemic control, and cardiovascular risk profiles in patients with T2DM. Future research should explore its applicability across diverse populations and investigate the underlying molecular mechanisms driving these improvements. Importantly, in cases where implementing both interventions is not feasible, exercise should be prioritized due to its broader and more robust impact across metabolic and lipoprotein outcomes.

## Figures and Tables

**Table 1:** Mean ± SD values of nutritional intake in the four study groups.

Groups	Energy (kcal/d)	CHO (g/d)	Fat (g/d)	Protein (g/d)
CG	2383.1 ± 106.6	305.1 ± 36.1	77.0 ± 15.7	117.3 ± 17
SG	2385.6 ± 157.0	304.6 ± 34.8	76.9 ± 14.2	118.7 ± 19.2
TG	2340.3 ± 213.4	291.6 ± 29.5	76.9 ± 14.5	117.0 ± 18.9
TSG	2358.0 ± 193.2	295.5 ± 32.4	79.1 ± 14.6	116.3 ± 17.8

CG: Control Group, SG: Supplement Group, TG: Training Group, TSG: Training + Supplement Group, CHO: Carbohydrate.

**Table 2:** Combined training protocols (aerobic + resistance exercise) for TG and TSG groups over 12 weeks.

Aerobic Training				Resistance Training			
Weeks	Durations (Min/Sessions)	Intensity (VO_2peak_)	Sessions (per Week)	Sets	Repetition	1-RM	Sessions (per Week)
1–2	10	60%	3	3	12–15	60–68%	3
2–4	15	65%	3	3	12–15	60–65%	3
4–6	20	70%	3	3	8–10	70%	3
6–8	25	70%	3	3	8–10	70%	3
8–12	30	70%	3	3	8–10	70%	3

Notes: 1-RM, one-repetition maximum.

**Table 3:** Body mass and BMI of the study groups.

	CG			SG			TG			TSG		
	Pre-Test	Post-Test	*p*	Pre-Test	Post-Test	*p*	Pre-Test	Post-Test	*p*	Pre-Test	Post-Test	*p*
Body mass (kg)	86.35 ± 3.34	86.74 ± 2.99	0.789	85.09 ± 4.93	85.89 ± 3.47	0.628	86.96 ± 4.39	79.69 ± 3.31	0.0007 *	86.67 ± 4.46	79.10 ± 4.86	0.0007 *
BMI (kg/m^2^)	28.34 ± 0.96	28.46 ± 0.96	0.777	27.39 ± 1.26	27.44 ± 1.58	0.947	28.51 ± 1.73	27.34 ± 1.41	0.065	28.07 ± 1.12	26.18 ± 1.83	0.021 *

Notes: BMI: Body Mass Index, CG: Control Group, SG: Supplement Group, TG: Training Group, TSG: Training + Supplement Group, * *p* < 0.05.

## Data Availability

The datasets generated and analyzed for this study are available from the corresponding authors upon request due to the privacy of the participants.
